# Preliminary Report on the Use of Metal Track on Railways as a Substitute for Wooden Ties

**Published:** 1889-08

**Authors:** 


### Preliminary Report on the Use of Metal Track on Railways as a Substitute for Wooden Ties, by E. E. Russell Tratman, C. E. Compiled by B. E. Fernow, Chief of Forestry Division, Department of Agriculture. Washington, D. C., 1889.

The continued and alarming waste of the forests in providing timber for use in the arts, and, let us also add, to create destructive and wasteful booms in our mountain streams, and the consequent rise in price of timber has induced railroad managers, who are among the largest consumers, to look about for some efficient substitute for wooden ties upon which the rails must rest.

Iron and steel are the materials looked to now to take the place of wood. Many patents have already been issued and some of the devices tried. The vast quantity of timber consumed for this purpose alone makes a demand upon our forests that has at last arrested the attention of political economists and has caused the Department of Agriculture of our own Government to issue a special bulletin (No. 3), the title of which appears at the head of this notice.

According to this bulletin, American railroad managers are not alone considering this subject. Iron ties are being tried in many countries of Europe, and even in Asia and Africa. In India the metal ties are being extensively used, and they have even been introduced into Burmah.

The number of patterns proposed is remarkable, no less than one hundred and sixty devices having been patented.

The most practical, according to the report, are the Post tie and the Durand tie. Both resemble inverted troughs in shape, but the Durand is made from old steel rails which are rolled into flat sheets and then bent into the proper shape. This reduces the cost of the metal tie to nearly that of wood.

We have not space to give more than the above summary of this report, and think that what is here stated is all that will interest the medical profession. W. M. J.

